# A Neurodynamical Model of Brightness Induction in V1

**DOI:** 10.1371/journal.pone.0064086

**Published:** 2013-05-22

**Authors:** Olivier Penacchio, Xavier Otazu, Laura Dempere-Marco

**Affiliations:** 1 Computer Vision Center, Computer Science Department, Universitat Autònoma de Barcelona, Barcelona, Spain; 2 Department of Information and Communication Technologies, Center of Brain and Cognition, Universitat Pompeu Fabra, Barcelona, Spain; University of Montreal, Canada

## Abstract

Brightness induction is the modulation of the perceived intensity of an area by the luminance of surrounding areas. Recent neurophysiological evidence suggests that brightness information might be explicitly represented in V1, in contrast to the more common assumption that the striate cortex is an area mostly responsive to sensory information. Here we investigate possible neural mechanisms that offer a plausible explanation for such phenomenon. To this end, a neurodynamical model which is based on neurophysiological evidence and focuses on the part of V1 responsible for contextual influences is presented. The proposed computational model successfully accounts for well known psychophysical effects for static contexts and also for brightness induction in dynamic contexts defined by modulating the luminance of surrounding areas. This work suggests that intra-cortical interactions in V1 could, at least partially, explain brightness induction effects and reveals how a common general architecture may account for several different fundamental processes, such as visual saliency and brightness induction, which emerge early in the visual processing pathway.

## Introduction

Brightness induction (BI) is the modulation of the perceived intensity of an area by the luminance of surrounding areas. BI provides a striking demonstration that visual perception of a given stimulus does not only depend on purely sensorial information (*i.e.* light) reaching the retina from such a stimulus but also on how the light is spatially distributed in its surroundings. Although early visual cortical areas are traditionally associated with the encoding of surface boundaries, their role in surface perception, and thus luminance perception, is still a matter of debate [1], [2]. The study of BI, which has been thoroughly investigated from a psychophysical perspective, offers an excellent opportunity to investigate the neural mechanisms that underlie brightness perception and the role of early visual cortical areas in such processing. To this end, computational neuroscience may prove an invaluable asset in bringing together psychophysical and neurophysiological experimental evidence with theoretical models that help establish links between them.

As reviewed in [3]–[5], the visual system processes information at different levels of complexity, which can be broadly classified into low-level, mid-level and high-level vision processes. The low-level approach to brightness perception finds its origin in Ewald Hering’s view, whereby adaptation and local interactions were regarded as crucial mechanisms at a physiological level. In contrast, the high-level approach finds a clear association with Hermann von Helmholtz’s view. He considered visual perception as a product of unconscious inference that occurs when our visual system performs its best guess as to what is in the visual scene. Following this view, both the sensory information but also prior experiences constitute the basis of the perceptual process, and BI would merely be a byproduct of the inferential process.

As will be demonstrated throughout this work, we conclude, in agreement with previous works (*e.g.* [6]–[10]), that a low-level approach can go a long way in accounting for BI phenomena. We furthermore propose a neurodynamical model that explains the mechanisms underlying such phenomena. The model takes into consideration the experimental evidence reviewed in the next sections. This model is therefore grounded on a vast body of work that is also critically revised herein. Thus, we first review several widely reported psychophysical effects that are crucial to unveil basic properties of BI, then the literature on neurophysiological correlates of BI, and, finally, a selection of computational approaches modeling the neural activity in the areas previously found to be relevant (*i.e.* mainly V1), and which are at the core of the model proposed in this work.

### Psychophysical Evidence Unveiling Fundamental Aspects of BI

In this section we review several psychophysical effects that reveal important aspects about the nature of the processes underlying BI. These effects are subsequently taken into consideration to assess the behavior of the proposed neurodynamical model. As will be discussed later, different models have successfully reproduced a broad variety of BI effects. Our motivation to investigate a neurodynamical model of BI is to both reproduce an ensemble of effects and scrutinize the neural mechanisms underlying them. Moreover, we address this challenge such that our modeling effort can be embedded in a global framework on visual information processing in the brain.

**Figure 1:**
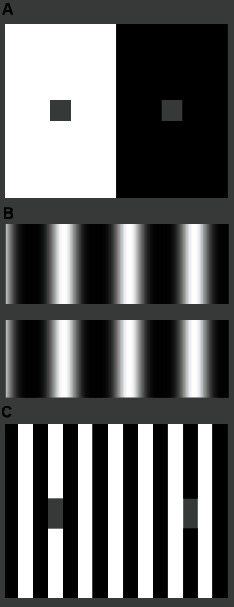
Illustration of some brightness induction effects. (**A**) Simultaneous brightness contrast (SBC), (**B**) grating induction (GI), and (**C**) White effect.

Commonly, BI effects are classified according to the perceptual direction of change, that is whether the change in brightness of the visual target departs from that of the surroundings (*i.e.* brightness contrast [11]) or otherwise tends to approach it (*i.e.* brightness assimilation [12]). One of the oldest known examples of brightness induction is the simultaneous brightness contrast (SBC) effect [11]. SBC is usually described as a homogeneous change in the brightness of a gray patch, which looks darker when located on a white background than a gray patch of the same luminance on a black background (see Figure 1**A**). A common explanation for SBC, grounded in the filling-in tradition, is that the brightness of the patch is determined by the information at its edges and is subsequently filled-in to the internal area. A second well-known example of brightness induction is the so called grating induction (GI) effect, an effect that produces a spatial brightness variation (*i.e.* a grating) across an otherwise homogeneous gray patch [13] (see Figure 1**B**). Not surprisingly, homogeneous brightness filling-in cannot account for GI. Accordingly, several brightness models have been proposed that incorporate non-homogeneous filling-in mechanisms [10],[14]. Nonetheless, it has been argued that these two phenomena (SBC and GI) may just be manifestations of the same underlying mechanisms [6] since, in fact, both of them constitute examples of brightness contrast.

Another well known BI effect is the White effect, whereby equiluminant gray test patches placed on top of either black or white bars in a square grating appear to have different brightness (see Figure 1**C**). Interestingly, a key aspect driving this effect is the contrast with the bar upon which the test patch is situated by effectively determining its immediate neighborhood. The White effect can thus switch from assimilation to contrast depending on the actual spatial configuration, which further suggests that assimilation and contrast also share the same underlying mechanisms [6],[8],[15].

No further examples of BI are introduced in this section (although some others will be reported in the Results section to demonstrate the potential of the proposed model) since the most basic phenomenological aspects regarding a common substrate for assimilation and contrast are already addressed in the examples considered here. The computational model developed in this work will propose a unified and biophysically inspired mechanistic to account for all of these aspects.

### Previous Computational Models of BI

Some of the most successful computational models of luminance perception were developed using multi-scale approaches to low-level vision. By considering operators emulating responses similar to those found in the receptive fields of neurons in early vision areas [16], they can account for a variety of effects [7]–[9]. The main differences between the models derive from the way the operators interact with each other and whether they are are combined with a contrast-sensitivity-function (CSF) (see [9] for a review).

This is the case for the unified brightness model based on difference of Gaussians (DOG) filters (*i.e.* low-level isotropic filters) originally proposed by Blakeslee and McCourt [6]. The authors later extended this model [7] by non-linearly pooling oriented differences of Gaussians (ODOG) (*i.e.* anisotropic filters) and adding a normalization procedure to equalize the global response at each orientation. Robinson et al. [17] later constrained the normalization to make it more neurally plausible and, as a consequence, were also able to reproduce more illusions. These models share with the Kingdom and Moulden’s MIDAAS [18] a number of features (*e.g.* spatial scale filtering and combining inputs across scales) but add the presence of more spatial frequency filters and a weighting scheme adjusted to match psychophysical data instead of making use of a set of rules.

Similarly, D’Zmura and Singer [19],[20] developed another multiresolution perceptual model in which the contrast of the surround was introduced by means of the so-called spatial pooling of contrast. By also making use of a multiscale and multiorientation approach, Otazu et al. [9] hypothesized that brightness induction was performed mostly between features of similar spatial frequency and orientation. The model considered a psychophysically determined CSF that explicitly included the observation distance. One of the most significant merits of the model was that it allowed the unification of brightness assimilation and brightness contrast in a single mathematical framework and, importantly, using a unique set of parameters.

The success of this type of model in capturing the main characteristics of BI phenomena strongly suggests that accounting for the operations performed by filters akin to early stage cortical neuronal receptive fields (*e.g.* multiscale spatial frequency and orientation selectivity, and normalization) is of utmost importance. Thus, although admittedly, higher-order visual processes might also play a role in luminance perception, the results of these models (as discussed by [7]) argue persuasively against the need of invoking higher-order inferential processes to explain the mechanisms underlying BI effects. Furthermore, the low-level approach offers an appealing connection between physiology and psychophysics, which has in turn been at the basis of most computational models of BI (including ours).

A different approach to tackle BI modeling attempts to build perceptual representations that keep the geometric structure of scenes. To this end, Pessoa et al. [10] proposed a network model that uses contrast-driven and luminance-driven representations, from which boundaries are extracted, and the neural activity is spread within “filling-in” compartments. This model produces a one-dimensional response profile that is assumed to be isomorphic with the percept. The “filling-in” is governed by a diffusive process, whereby boundaries act as gates of variable resistance to diffusion, and takes place before the contrast and luminance signals are recombined to deliver the output of the model. The network model includes the notions of simple and complex cells responses (with ON and OFF channels for simple cells) and, importantly, feedback competition between complex cells. Being grounded on Grossberg’s work (*e.g.* [14],[21]), it can be allocated within a larger framework that addresses biological vision in a broader context than luminance perception.

In contrast to the previous approaches, which attempt to model different aspects of human physiology or perception, Corney and Lotto [22] used artificial neural networks to emulate the process of experiential learning from stimuli with feedback from the environment. These authors, thus, posit their model within the framework of visual ecology. Their results suggest that “illusions” (which include BI effects and are reframed in their work as the condition in which the true source of a stimulus differs from what is its most likely, and thus perceived, source) arise because (i) natural stimuli are ambiguous, and (ii) this ambiguity is resolved empirically by encoding the statistical relationship between images and scenes in past visual experience. Interestingly, a recent study presented by Coen-Cagli et al. [23] relates the computational and ecological principles underlying contextual effects and suggests that the influence of the context on a target stimulus is determined by their degree of statistical dependence. This provides a link between the two approaches and, as will be stressed later, lends statistical support to the theory that V1 computes visual saliency, a notion that is closely related to principles of the model that we propose for explaining BI effects.

As emerges from this introduction, BI offers an experimental paradigm that can be employed to investigate fundamental aspects of visual information processing in the human visual system. To this end, we propose a biophysically-based neurodynamical model of BI which, in contrast to most of the previous models, also accounts for the dynamical evolution of the system, thus allowing to explore dynamical stimuli and to probe fundamental aspects of visual information processing in early vision. Although this is a feature that is shared with the network model proposed by Pessoa et al. [10], it is worth noting that they only consider one-dimensional static stimuli.

### Neurophysiological Correlates of BI

Although striate cortex is traditionally regarded as an area mostly responsive to sensory (*i.e.* retinal) information, neurophysiological evidence suggests that brightness information might be explicitly represented in V1. Such evidence has been observed both in anesthetized cats [2],[24], where neuronal response modulations have been found to follow luminance changes outside the receptive fields (RF), in macaque monkeys [25], and in human fMRI measurements [26]. EEG recordings further support that brightness perception correlates with early activity in the striate cortex, suggesting that induction phenomena are essentially bottom-up [5].

In particular, Rossi and Paradiso [24] reported brightness changes in the receptive field (RF) of V1 cells covered by uniform gray illumination when the luminance of rectangular flanking regions was modulated sinusoidally in time. They found that the responses of retinal ganglion cells never correlated with brightness whereas many neurons in striate cortex and a small fraction in the LGN responded in a phase-locked manner at the temporal frequency of the flank modulation. This was the case even though the flanks were 3–7° beyond the edges of the RF, thus providing experimental support for the hypothesis that brightness information, and not just contrast, is explicitly represented in the responses of neurons in V1. This supports the view that neural representations of object surfaces exist already in V1 and suggests that lateral interactions, known to play an important role in mediating contextual effects (*e.g.* [27],[28]), may also underlie BI. In contrast, van de Ven et al. [1] report a stronger correlate of BI in fMRI measures in V2 than in V1. However, the authors also claim that their results do not exclude a possible contribution of V1 to brightness perception.

Thus, recalling all of this converging evidence suggesting a relevant role of V1 in brightness perception and hence, in BI, the neurodynamical model proposed in this work focuses in V1 and accounts for the contextual effects therein occurring, which we hypothesize are at the basis of BI. In the next section, we review previous computational models of V1 which have addressed contextual effects from different perspectives and with different aims.

### Computational Models of V1 Neuronal Activity and Contextual Effects

The notion of contextual influences on visual processing is broad since it might refer to the effect that stimuli present at different points in space or time have on other stimuli, but may also involve more complex constructs such as attention or memory [29]. In this paper, we only consider contextual influences arising from interactions in which information in one spatial region of the visual image affects the interpretation of another region.

Although higher cortical areas may undoubtedly play an important role in the processing of contextual effects through modulatory feedback, some phenomena may also be explained by the dynamic interplay between feedforward projections and horizontal intracortical connections in V1. Series et al. [30] review experimental and theoretical progresses in the description of the so-called “Center/Surround” modulations and their neural basis. To this end, they distinguish three different types of models: (i) *phenomenological* models which aim at characterizing the response properties within the context of a visual information processing algorithm, (ii) *structural* models which aim at characterizing the biophysical neural mechanisms that are responsible for the physiological data, and (iii) *optimized* models that try to predict the physiological data from an optimized strategy of visual coding.

In this work, we are interested in the structural approach while we also try to establish links with phenomological approaches which have been previously employed to investigate BI. However, it is worth pointing out that studies such as the one presented by Coen-Cagli et al. [23] contribute to build bridges between the three approaches.

Central to the work presented in this article is the neurodynamical model of V1 originally proposed by Li [31] to explain contour integration. This model has also been successfully applied to explain several contextual effects such as figure–ground and border effects [32], visual saliency [33] (and other related ones such as pop-out and asymmetry in visual search [34]), and preattentive segmentation [35]. It largely relies on local intra-cortical interactions mediated by horizontal connections to reproduce all of these effects. It has gained convincing experimental evidence (both neurophysiological and psychophysical, *e.g.* [36],[37]) regarding one of its main predictions, *i.e.* the existence of a saliency map in V1. Notably, the results reported by Coen-Cagli et al. [23] lend further statistical support to the theory that V1 computes visual saliency, a strong prediction of Li’s model. In later sections (Results and Methods, as well as in Texts S1–S4), a full account of this model will be presented.

## Results

As previously stated, to understand the neural basis of BI, a minimal (although biophysically-inspired) computational model of visual object representation in V1 is considered. The proposed model is based on that introduced by Li [31],[35], which was originally developed to investigate the neurodynamical basis of contour integration and pre-attentive visual segmentation in V1. To the best of our knowledge, the modeling effort presented in this work constitutes a new approach to BI which tackles directly its neurodynamical basis. Thus, although Li’s model itself has been extended in two ways (later discussed), it is the overall framework proposed in this work which mainly conveys the novelty.

**Figure 2:**
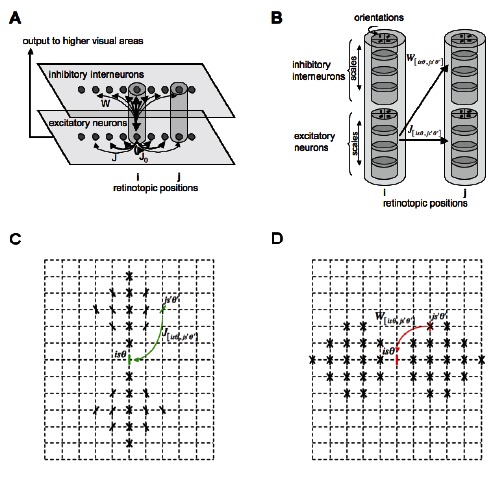
Schematic of the V1 model. (**A**) The visual input is sampled in a 2-dimensional discrete regular grid (here reduced to a single dimension for the sake of clarity). At each point on the grid, an ensemble of units representing neural populations sensitive to different spatial frequencies and orientations regularly distributed within the interval 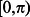, and which share the same receptive field center, is located, thus emulating a V1 hypercolumn. A population of excitatory neurons in one hypercolumn can interact with another excitatory population in another hypercolumn both through monosynaptic excitation 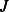 or though disynaptic inhibition 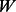. A population of excitatory neurons in one hypercolumn can interact with itself through self-excitation 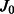. The output of the layer of excitatory units is sent to higher visual areas. (**B**) Each hypercolumn is composed of a set of excitatory and inhibitory cells tuned to different spatial frequencies (*i.e.* scales) and orientations depicted in the sketch. A neuron population sensitive to a given spatial frequency 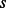 and orientation 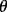 in the hypercolumn at retinotopic position 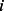 interacts with another neuron population sensitive to spatial frequency 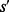 and orientation 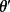 in the hypercolumn at retinotopic position 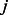 both directly through monosynaptic excitation 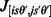 and disynaptic inhibition 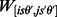. Panel (**C**) sketches the weights of the excitatory connections 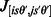 to the postsynaptic unit 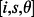, and (**D**) sketches the weights of the inhibitory connections 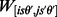 to the unit 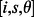. Both 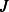 and 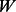 are translation invariant.

As in Li’s original model (described in detail in the Methods section and in Text S1), visual stimuli are characterized as neuronal signals at discrete spatial locations. At each of these locations, a V1 hypercolumn is composed of 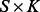 unit pairs (one excitatory and and one inhibitory unit per pair), as illustrated by the schematic representation of the network architecture shown in Figure 2**A**. Each unit within a hypercolumn is characterized by a triplet [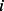, 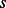, 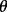], where 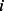 is the RF center, 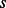 is the preferred spatial frequency, for 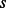 = 1, 2,…, and 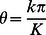 is the preferred orientation, for 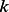 = 1, 2,…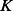. Note that each unit in the model corresponds to a mathematical abstraction of a local neuronal population formed by cells of the same type and selectivity.

The response of the model is mainly determined by both its input image 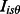 (or sequence of input images 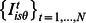), and the interactions between the different neuronal populations. In particular, a unit in one hypercolumn can interact with another unit in a different hypercolumn both via monosynaptic excitation through the excitatory-excitatory horizontal connections described by 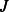, and disynaptic inhibition via the excitatory-inhibitory connections described by 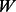, as sketched in Figure 2**B**. The matrices 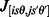 and 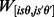 are the key terms of the model since they strongly determine the dynamical behavior of the network. The matrix 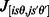 indicates how the neural activity of excitatory unit 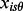 at position 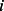, scale 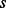 and orientation 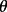 is related to the neural activity 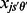 at position 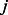, scale 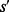 and orientation 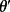. As in [35], the monosynaptic excitation 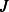 is strong for cells at neighboring positions which have similar orientations and are coaligned, thus leading to a connectivity pattern with a typical bow-tie shape since 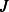 predominantly links cells with aligned RFs for contour enhancement. In contrast, 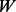 mainly links cells with non-aligned RFs for surround suppression. The structure of both 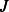 and 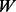 are sketched in Figure 2**C,D** for any two neuronal populations selective to the same (or similar) scale.

Regarding the modifications introduced in the original model [35], on one side, our model considers a complete multiscale and multiorientation wavelet decomposition of the visual stimuli, thus allowing for the processing of arbitrary images rather than just the simple edge models originally considered [31],[35]. On the other side, we have further introduced scale interactions in the model by establishing connections between neuronal populations with different preferred spatial frequencies. The existence of connections of such kind is in agreement with psychophysical experiments [38],[39] and was also predicted by [40]. We hypothesize that the effect of scale and orientation can be modeled independently such that 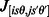 (similarly, 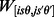) can be written as the product 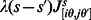 (similarly, 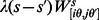), where 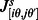 (similarly, 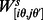) is akin to the monosynaptic excitation (dysynaptic inhibition) term in the original model (see Text S1), and 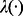 is a symmetric Gaussian-like function that peaks at 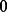 and decreases rapidly.

In the model, the excitatory and inhibitory cells have membrane potentials 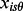 and 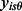, respectively, and their outputs are obtained from sigmoid-like positive non-linear and non-decreasing functions 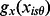 and 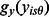, which represent the firing rates. The membrane potentials obey the following differential equations for an input image chacterized as 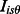:
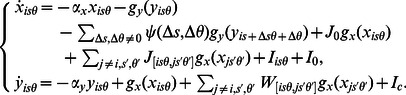
(1)


Further details about the model including the explicit mathematical expressions of the synaptic coupling strengths 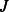 and 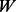 and the rest of undefined terms are presented in the Methods (or Text S1). As for the visual input (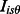), in this work, both static and dynamic stimuli have been considered. Whereas static stimuli have been broadly studied in psychophysics and have yielded a large body of well-characterized BI effects (some of which have been previously reviewed), dynamic stimuli (herein, temporal modulations of surface luminance) are gaining increasing interest because they can be directly correlated with neurophysiological measurements. In the case of static stimuli, the input to the network follows the complete wavelet decomposition previously presented in [9] (see also Text S2). In contrast with the classical Gabor decomposition, which is often used to model the receptive fields of V1 cells [16], the wavelet decomposition has an inverse transform, which allows us to build a perceptual image. Thus, the visual stimulus 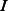 is decomposed according to the following formula:
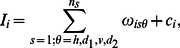
(2)where 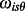 correspond to the multiresolution planes, 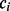 is the residual plane, 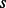 denotes the spatial scale, 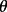 represents the orientation (*i.e.* 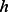 horizontal, 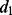 first diagonal, 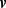 vertical, and 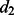 second diagonal), and 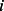 means the 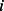-th multiresolution coefficient and establishes its spatial location. The coefficients 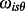 are derived from a multiscale and multiorientation wavelet decomposition which separates the (achromatic) image into different spatial frequency and orientation components (reminiscent of striate single-cells receptive fields).

For a given input image, the perceptual image reflecting the perceived intensity, or brightness, of a static stimulus is recovered using:
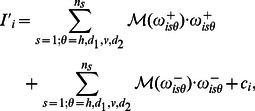
(3)where 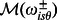 is the temporal average of the model output over several oscillation cycles. Depending on the visual stimuli, the system settles into an oscillatory steady state, and temporal averages of 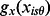 over several oscillation cycles (*i.e.* 12 membrane time constants [35]) are used as the output of the model.

When recovering the perceptual image, 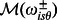 effectively acts as a weighting function of the wavelet coefficients while, in turn, it depends on such coefficients. Since the polarity of contrast information must be preserved in order to enable the reconstruction of the perceived image, a separation into positive and negative coefficients (reminiscent of the separation into ON and OFF channels found in LGN and thoroughly discussed in previous computational models [10],[21]) has been considered (*i.e.* 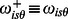 if 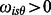, and 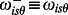 if 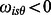).

In the dynamic case, the modulation of luminance in the visual display is seen as a sequence of image frames 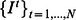. Each frame is decomposed in the same way as a static stimulus, which leads to sequences of multiresolution coefficients 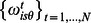 and residual planes 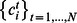. The expression for a sequence of perceived images in the dynamical context is equivalent to that obtained for static stimuli.
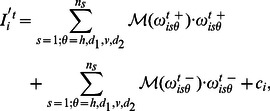
(4)where, however, 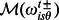 corresponds to the direct output of the excitatory cells without averaging over time, namely 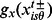. A thorough description of the parameters of the model is given in Text S1.

### Static Stimuli

In this section, we apply the model to the classical effects of static BI outlined in the introduction and analyze the results obtained. Special emphasis is paid to the mechanisms underlying such results. Moreover, in order to explore the potential and scope of the proposed model, we further consider an ensemble of different BI effects widely reported in the literature and compare them with the results predicted by our model.

#### Simultaneous brightness contrast

**Figure 3:**
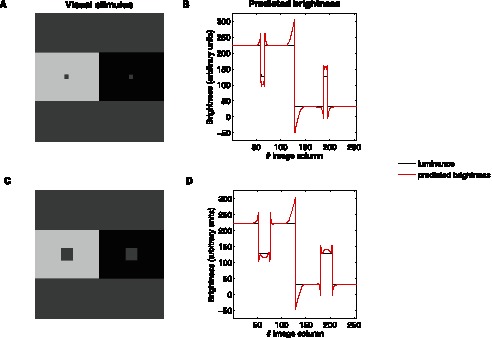
Simultaneous contrast effect. (**A**,**B**) Examples of the simultaneous brightness contrast (SBC). (**B**,**D**) Model prediction. Here, and in the following figures showing brightness profiles, *visual stimulus* (black solid curve) refers to the profile of the luminance stimulus and *brightness* (red solid curve) corresponds to the profile of the perceived luminance as predicted by the model. According to the model, the gray rectangle is perceived darker when it is surrounded by a bright background and brighter when it is surrounded by a dark background, in agreement with perception.

Figure 3**A,C** shows two different instantiations of the SBC effect, corresponding to two different scales. In both cases, the gray patch is predicted to be perceived darker when it is surrounded by a bright background and ligther when it is located on a dark background. In Figures 3**B,D**, we see that the model correctly predicts the changes in brightness of the gray patch. The underlying mechanisms explaining why the model predicts SBC can be summarized as follows. Consider the gray patch on the right side of the stimulus. The neural population sensitive to its spatial frequency will show a strong response. Note that all the orientations are equally represented here, so the analysis is valid for any of them. Very locally, since few model cells respond to the patch, excitatory cells of the same type mutually contribute to the activity of each other by virtue of monosynaptic excitation 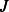. On the other hand, in the area surrounding the patch there is no similar feature with non-zero contrast at the same frequency. Accordingly, around the gray patch the activity of the excitatory cells sensitive to similar orientations and frequencies is very low. This causes the inhibition of the excitatory cells responding to the patch to be low since inhibition is mediated by the neighboring excitatory activity through disynaptic inhibition 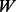. Thus, there is an increase in the excitatory activity of the population sensitive to the spatial frequency of that patch. The modification of the initial activity of the excitatory cells produced by the visual input through the recurrent interactions thus results in brightness contrast. This effect can be considered as a direct illustration of the workings of Li’s model, which is designed to uncover disruption in input homogeneity. It is worth pointing out that, since we have introduced a mechanism which allows the consideration of a wide range of spatial frequencies, the model is able to detect disruption at very different scales, as illustrated by Figure 3**D**. The preceding reasoning is independent of the polarity of the contrast (as long as the separation between the two polarities is kept) as illustrated by the fact that the increase in the activity of the excitatory neural populations responding to the spatial frequency and orientations of the left patch also results in brightness contrast and produces a decrease in the perceived luminance with regard to the original luminance of the gray patch on the right.

#### Grating induction effect

**Figure 4:**
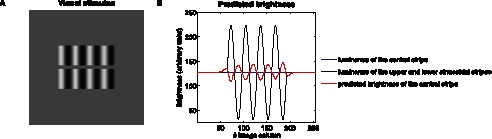
Grating induction effect. (**A**) Example of the grating induction (GI) effect. (**B**) Model prediction. The panel shows the original luminance of the horizontal stripe (black dashed curve) and the brightness predicted by our model (red solid curve), which changes in counterphase to the upper and lower horizontal sinusoidal luminance gratings (black solid curve), in agreement with psychophysical observation.

Figure 4**A** shows an illustration of the GI effect. The central horizontal patch between the two sinusoidal gratings has constant luminance but is perceived as a sinusoidal grating in counterphase to the upper and lower sinusoidal stripes. Figure 4**B** shows that the alteration of the luminance is also correctly predicted by the proposed model. This chiefly arises because any location on the central patch between two depressions (resp. peaks) of the upper and lower stripes produces a strong activity in the neural population sensitive to its spatial frequency and orientation (here, mainly horizontal) as a consequence of the difference in luminance. This occurs for both contrast polarities (*i.e.* white-gray-white and black-gray-black). Since there are no similar features at the same frequency in the surrounding area, and similarly to the case discussed above for SBC, there is an increase in the excitatory activity of the population under consideration and virtually no inhibition from the neighboring units (defined locally by the spatial locations covered by the connectivity matrix 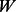), this causes the mean firing rate of these units to increase and induces contrast with respect to the two depressions (resp. peaks), resulting in the sinusoidal profile in counterphase to the upper and lower sinusoidal stripes.

#### White effect

**Figure 5:**
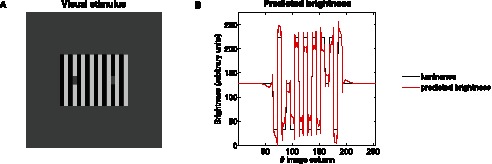
White effect. (**A**) Example of the White effect. (**B**) The mean firing rate predicted by the model differs at the two locations corresponding to the two rectangles (around columns 80 and 170 of the image, respectively) whose (physical) luminance is equal, in agreement with perception.

Figure 5**A** provides an example of the White effect whereby the gray rectangle on the left is perceived darker than the one on the right. Figure 5**B** shows how the proposed neurodynamical model reproduces the White effect. The main features characterizing the overall operation of the model can be described as follows. Consider the gray patch on the left side of the stimulus. For this gray patch, the strongest neural responses that can be encountered will, of course, correspond to those populations selective to the spatial frequency corresponding to the size of the patch and whose receptive fields have either a vertical orientation (for the black-gray-black transition along the horizontal direction) or, to a lesser extent, a horizontal orientation (for the white-gray-white transition along the vertical direction). Importantly, the neural responses of nearby populations selective to the vertical orientation are, in fact, even stronger, when the background grating is considered since it shares with the patch the same spatial frequency and vertical orientation and has more contrast, thus resulting in a strong excitatory activity in its surround. Hence, there is a strong contribution to the inhibitory activity through disynaptic inhibition 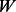, which causes the excitatory activity of the units responding to the patch to decrease. Accordingly, the model implements assimilation of the patch brightness by that of the dark vertical stripes in lateral contact. Moreover, regarding the white-gray-white transition along the vertical direction, the situation is equivalent to that found for the SBC and GI effects in that brightness contrast is implemented and the overall perceptual direction of change results in a lowering of the brightness. The same explanation holds for the patch on the right side by simply inverting the polarity of the transitions.

#### Mach bands

**Figure 6:**
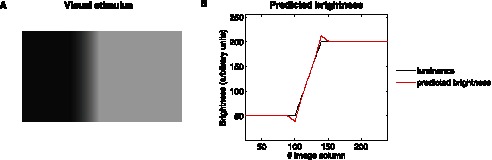
Mach bands effect. (**A**) A dark band and a bright band appear at the edges of regions whose luminosity is constant. (**B**) The mean firing rates predicted by the model agree with the illusion that there are two conspicuous bands at the edges between the ramp and the plateaus.

The phenomenon known as Mach bands corresponds to the perception of a bright and a dark band at both sides of a ramp between two plateaus whose luminance is constant (see Figure 6**A**). In Figure 6**B**, we show that the model is also able to reproduce this effect. To understand the main mechanisms of the model responsible for this effect, the edge on the right side of the ramp will first be considered. As a consequence of the ramp, a diffuse boundary (edge) with vertical orientation emerges. Thus, the neurons mostly sensitive to such an orientation will exhibit a strong activity when compared to the rest of neurons sensitive to different orientations in the same hypercolumn. Furthermore, the cells which are aligned along all the locations corresponding to such a diffuse edge, and sharing the same orientation sensitivity, will benefit from an enhanced activation as a consequence of the recurrent connections derived from the connectivity matrix 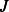. Since no further colinear features (*i.e.* nearby edges with the same contrast polarity) are present in the stimulus, the inhibitory activity onto these excitatory cells, as mediated by 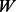, will become negligible. As a consequence, an increase in the excitatory activity of the neuronal cells colinear to the diffuse edge will take place, thus giving rise to the emergence of a bright band. A similar reasoning explains the dark conspicuous band on the left of the ramp when the opposite polarity is considered.

More results are provided in Text S3. In particular, we address the influence of relative orientation on BI and, importantly, come up with a prediction on its effect (see Figure S1 in Text S3). We also consider the Chevreul effect (see Figure S2 in ).

### Dynamic Stimuli

**Figure 7:**
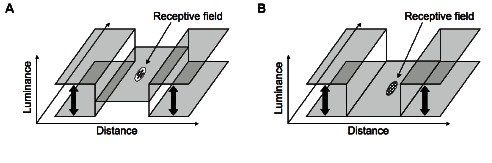
Spatial configuration of the dynamic stimuli. (**A**) The stimulus is composed of three equally-sized rectangular regions. The luminance of the two areas flanking the central gray area is modulated sinusoidally in time (as indicated by the thick black arrows) whereas the static center region of the stimulus had a luminance equal to the time-average luminance of the modulated flanks. (**B**) The stimulus is similarly composed of three equally-sized rectangular regions, the luminance of the two flanking areas is modulated as described in (**A**), but no light is shed on the central region, and hence on the indicated receptive field. As observed in [24], the brightness of the static central area varies in counterphase to the flanks when the corresponding neuron population has induced activity from its own receptive field (**A**) but not when this activity is null (**B**).

We have so far reported the results obtained for a number of BI effects when static stimuli are considered. Nonetheless, one of the main strengths of the proposed model is that being a dynamical model it provides a full description of the temporal evolution of the system, which is of utmost interest when considering dynamical stimuli. Thus, we further tested our hypothesis that contextual influences in V1 play a central role in luminance perception in a dynamical context. As reviewed in the Introduction, Rossi and Paradiso [24] reported brightness changes in the activity of neurons in V1 even though no modulations of luminance were induced in their classical receptive fields. Figure 7**A,B** illustrates the kind of stimuli used by Rossi and Paradiso. In Figure 7**A**, a central band was covered by a uniform gray illumination. Some white and dark bars were added over a central receptive field. The luminance of the central gray part and that of the white and dark bars were held constant. The luminance of flanking regions was modulated sinusoidally in time keeping their mean luminance at the same level of the central uniform gray band. They reported that for low temporal frequencies of the sinusoidal modulation, a large population of neurons in striate cortex responded to luminance variation beyond their receptive fields. In addition, these neurons responded in a phase-locked manner, namely in antiphase to the flanks, at the temporal frequency of the sinusoidal modulation of the flank luminance, even though the flanks were 3–7° beyond the edges of the receptive fields. There was, however, no perceived modulation of the activity when no light reached the classical receptive field of the neurons, as in the stimulus shown in Figure 7**B**.

**Figure 8:**
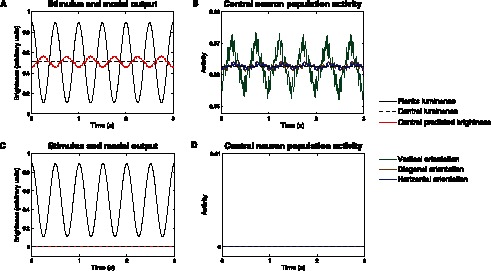
Response to the dynamic stimuli. (**A**) Output of the model in response to the stimulus outlined in Figure 7**A**. The black solid curve describes the sinusoidal oscillations of the luminance of the flanks, the black dashed curve represents the luminance of the central part of the stimulus and the red solid curve gives the brightness of this central part, according to the model. The model agrees with the perception that the brightness of the static central area varies in antiphase to the flanks. (**B**) shows the modulation of the firing rate of neuron populations sensitive to the highest frequency and located in the center of the stimulus due to the modulation of the flank luminance. The modulation of the flanks luminance takes place outside their classical RF since the two flanks are separated by 24 pixels (or units) and the diameter of the RFs of these neurons is 5 pixels. The induced modulation is high for the population of neurons that share their preferred orientation with the neurons that best respond to the edges created by the flanks, namely the vertical orientation (green solid curve), and is low if the difference between orientations is high, as for the diagonal (brown solid curve) and horizontal (blue solid curve) orientations. (**C**,**D**) Output of the model in response to the stimulus outlined in Figure 7**B**. According to the model, induction is lost when there is no inherent activity in the central part of the stimulus, in line with the observations described in [24].

We investigated the response of our neurodynamical model to Rossi and Paradiso’s stimuli. Figure 8**A** shows that the model accounts for luminance changes outside the classical receptive field. Specifically, the firing rate of the population of neurons sensitive to high spatial frequencies whose receptive fields are centered in the central uniform gray band shows sinusoidal activity. To assess the difference in phase between the luminance modulation in the flanks and the response of the neuronal population at the center of the central band, the neural response was time-convoluted with a sliding square weighting function with a period equal to the inverse of the modulation rate (as done by Rossi and Paradiso, cf. Figure 3 in [24]). The temporal frequency of the modulation coincides with that of the luminance of the flanks, but the response is in counterphase, in agreement with the experimental results reported by Rossi and Paradiso in [24]. It is worth noting that the flanks are at a distance of 2.5 receptive fields from the location of the neurons sensitive to the highest spatial frequency (Figure 8**B**).

The underlying mechanisms explaining why the model replicates the counterphase modulation of the brightness can also be understood by paying special attention to the dynamical evolution of the inhibition between neighboring neural populations sensitive to similar spatial frequencies and orientations. In Figure 7, the neural population with receptive fields showing a preference for vertical edges and the highest spatial frequencies is inhibited by the neural population responding to the edges emerging from the modulation of the luminance in the flanks. The higher the luminance variation, the more pronounced will be the edges, thus leading to stronger neuronal responses (*i.e.* higher firing rates) at the boundaries of the flanks. Such activity will be fed back into the excitatory neurons at the central gray band as inhibitory currents thus inducing the counterphase effect encountered since the propagation of the signal is effectively immediate when compared to the low temporal frequency (*i.e.* 2 Hz) of the driving signal.

Interestingly, the inhibitory effects (and thus the modulation observed) in the central units decreases when the difference between the orientation of the edges created by the flanks and the preferred orientation of these units increases, as shown in Figure 8**B**. This phenomenon, which is a consequence of the structure of the connectivity matrices (see Figure 2(**D**)), is also a prediction of our model.

Finally, the influence of luminance modulation beyond the classical receptive field is removed when the neuron population at the central flank has no proper activity, as can be seen in Figure 8**C,D**, in line with Rossi and Paradiso’s experiment.

### Relation with Previous Models

As emphasized in previous sections, the main difference between the model presented in this work and other models of brightness induction lies in that, in addition to reproducing classical BI effects, it aims at apprehending plausible neuronal mechanisms underlying BI, for both static and dynamic induction phenomena. Thus, although gaining biological plausibility has an added cost (namely an increase in the number of degrees of freedom), it also carries some crucial advantages, *i.e.* a single model focused on the effect of contextual influences in V1 can account for several fundamental processes simultaneously, a feature that is highly desirable when modeling brain function. In addition, the parameters have not been chosen arbitrarily, but are essentially shared with Li’s model [35], and are based on available neurobiological evidence. A general description of the most successful computational models of BI is available in the introduction. It is worth noting, for instance, that another model in the literature has a similar focus [10], but is restricted to static one-dimensional stimuli. In this section, we concentrate on the resemblance of the model with two prominent models using a multiscale approach to low-level vision, namely the BIWaM and the ODOG model.

The mathematical formulation we adopt for constructing the predicted perceptual image is equivalent to that described in the BIWaM model presented in Otazu et el. [9]. However, in this last model, instead of using a term similar to 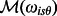 (*i.e.* the output from the proposed neurodynamical model) to mediate the reconstruction of the image, the authors consider the following function:
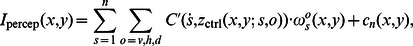
where 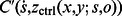 (see [9] for details) is built on the notion of CSF but is modified to account for the following three assumptions: (i) brightness assimilation is only performed when both central and surround stimuli have similar spatial frequencies within a frequency range of about one octave, (ii) brightness assimilation is strongest when the central stimulus and the surround stimulus have identical orientations, whereas for increasing relative spatial orientations brightness assimilation is weakest and brightness contrast is strongest, and (iii) when the luminance contrast of the surround features increases, brightness assimilation increases (*i.e.* brightness contrast decreases) and *vice versa*.

Since the proposed neurodynamical model shares with the BIWaM the wavelet decomposition, it makes sense to ask whether these assumptions can be accounted for as an emergent effect of the topological and the dynamical properties of our model. It turns out that the connectivity matrices 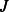 and 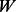 promote effects compatible with the second assumption since neurons that respond to similar orientations are more strongly connected than neurons responsive to rather different orientations. Furthermore, neurons responding to the same or one octave apart spatial frequencies are more strongly connected than those tuned to different spatial frequencies. Finally, one of the key aspects of the BIWaM model is the weighting that modulates the CSF employed in the reconstruction of the image by considering the relative contrast of a central feature compared to the contrast of its surround features. In particular, this is done by means of the variable 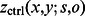 whose definition is based on the ratio 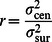, where 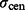 refers to the standard deviation of the filter responses to the central feature and 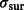 corresponds to that of the surroundings. In our model, such an effect is implemented dynamically by the cooperative and competitive processes taking place between the neurons at the central location (cen) and those located in the surroundings (sur). Furthermore, the non-linearity instantiated by the function 
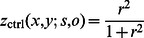
 finds its neurophysiological counterpart in the divisive normalization introduced through 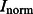.

The model proposed in this work also shares with the ODOG model two of its essential features, namely multiscale spatial frequency sensitivity and orientation specificity. It is also worth pointing out that the proposed model provides interesting insights into the cortical mechanisms underlying the Blakeslee and McCourt model [7]. In particular, we have conducted a study to reveal whether the proposed network reproduces an operation akin to the differential weighting discussed in [7]. To this end, several sinusoidal gratings characterized by different spatial frequencies were considered as input stimuli to the network and the gain between the output of the model and its input was analyzed. As a result, a gain function with a slope of 0.092 in log-log coordinates (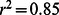, 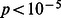) is obtained (see Figure S3 in Text S4) that resembles the power function with slope 0.1 that was used to weigh the filters considered by Blakeslee and McCourt model [7] and is consistent with the shallow low-frequency fall-off of the suprathreshold CSF [6] associated with the high-contrast stimuli employed throughout this last work.

## Discussion

This study reports the results from a computational investigation into the neuronal mechanisms underlying several BI effects observed either in human psychophysics [6],[9],[11],[13] or in neurophysiological recordings [2],[24]–[26], which have been obtained from different modalities and species. We further attempt to provide new evidence that sheds some light on the view that BI may, at least partially, be explicitly represented in V1. This is in contrast to the more common assumption that the striate cortex is an area mostly responsive to sensory information.

The proposed model has been built up on a minimal neurodynamical model of V1 [31] which has been successful in accounting for a number of other contextual effects (*e.g.* visual saliency, segmentation, contour-enhancement, etc). This study therefore suggests that BI shares with visual saliency and these other contextual effects a common neural circuitry. The proposed model inherits from Li’s model the topological structure of the network which grants a prevalent role to the horizontal connections. This, in fact, constitutes one of the strengths of the model as it is embedded in a larger framework aiming to explain other fundamental processes of biological vision and has found supporting experimental evidence both in psychophysics [41] and neurophysiology [36]. Thus, the study of BI, beyond being an exciting research topic on its own, leads to a scenario that can help reveal some fundamental aspects of visual information processing in the human visual system. It is with this motivation that we have addressed this investigation. Consequently, an effort has been made throughout this work to frame our results in a larger picture and relate them to other basic visual processes.

Notably, one of the main strengths of the proposed model is its biological motivation. In contrast to previous approaches (*e.g.* [6],[7],[9]), which emulate basic findings by imposing high-level constraints (*e.g.* contrast normalization, modulation of scale effects by means of a CSF, etc), these become emergent properties of the proposed network and are derived from well-known built-in cortical mechanisms and structural properties such as synaptic connectivity or divisive normalization. From a neuronal perspective, divisive normalization is a widespread computation in a variety of sensory systems [42]. Recent results clearly show that contrast sensitivity is continuously regulated and normalized over the whole activated V1 cortical surface by means of a dynamic normalization pool [43]. The proposed network model includes a normalization term (as discussed in [31],[35]), which in fact may be at the basis of the contrast normalization operation invoked by [7] to promote the equalization of the global response at each orientation. Interestingly, contrast normalization has been found to play a central role in explaining the transition from assimilation to contrast effects as, for instance, that found in the White effect [6],[7],[15].

Furthermore, by providing direct predictions of neural activities, the proposed neurodynamical model can be validated both in a direct way by means of neurophysiological measures but also in an indirect way by means of surrogate psychophysical percepts. In this sense, the fact that our model is able to reproduce the neurophysiological recordings reported in [24] provides strong support for its overall validity. This is complemented by the general agreement with perceptual data encountered when dealing with the static stimuli. Although it is beyond the scope of this study to address issues such as neural decoding in order to build visual percepts, what we claim with the reported inverse transforms is that the model outputs are compatible with the expected percepts. Accordingly, a simple inverse method is able to reproduce such percepts on the basis of the visual inputs and how these are modified through contextual effects.

As already pointed out, one of the unique features of this study is precisely that both static and dynamic visual stimuli have been investigated. This suggests that the mechanisms that have been identified are robust across stimuli and can be closely related to neural representations at realistic time scales. This follows from the fact that the model is a neurodynamical system that can naturally deal with any kind of visual stimulus, in contrast to most other successful formulations (*e.g.* [6],[7],[9]), which do not incorporate dynamical aspects.

It is worth noting that some of the existing models which account for dynamical effects, *i.e.* [10],[14],[21], do rely on the existence of filling-in signals. These theories suggest that surface brightness is represented explicitly by neural signals in cortical visual field maps, which are initiated by contrast signals at the stimulus borders. By using functional magnetic resonance imaging (fMRI) to search for such neural “filling-in” signals, Cornelissen et al. [44] found no evidence for these kinds of signals and concluded that the visual field maps of human V1 and V2 do not contain filled-in, topographical representations of surface brightness (and color). In contrast, our model reflects mechanisms for which converging experimental evidence exists [36],[42],[43],[45].

Since BI can be simply regarded as a contextual effect, recurrent networks of excitatory and inhibitory neurons provide a convenient framework for modeling. However, the specificities of the different BI effects can only be captured provided appropriate connectivity matrices are considered. It is worth noting, for instance, that some induction effects (e.g. SBC, Mach, or Chevreul, among others) can already be recovered by considering the raw gray level images as visual inputs to a simplified 1D version of the proposed model in which the synaptic connectivities are defined by means of unidimensional gaussian functions of different widths. In that case, the behavior of the interactions that 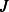 and 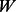 implement is akin to a center-surround mechanism. However, when 2D stimuli such as arbitrary images are considered, orientation is likely to become relevant and raw gray level values can no longer be used. Instead, Gabor-like filter responses and connectivity matrices akin to those reported in this study must be considered. Although Li [35] already suggested that this should be done, one of the contributions of this work is that we have effectively implemented this notion by considering a complete wavelet-decomposition that makes use of a set of filters similar to a Gabor filter bank, thus keeping a biological substrate, while allowing for the reconstruction of a perceptual image on the basis of the modified output for validation purposes.

Moreover, our model also includes scale selectivity and interactions between the scales. Neither of these aspects were considered in Li’s original model [31],[35]. On the one hand, by including scale selectivity, we have been able to account for BI effects that occur at different spatial scales (see for instance the SBC reported in the Results section). On the other hand, although not critical to reproduce the reported results, the existence of scale interactions has been suggested by psychophysical experiments [38],[39] and also predicted by [40].

Throughout this investigation, a close analysis of the mechanisms underlying the reported BI effects has revealed that inhibition is of utmost importance to enable the emergence of the observed perceptual effects. Interestingly, this is an observation that coincides with the prevalent role that inhibition has long been acknowledged to play in modeling other cognitive processes such as working memory [46] or decision making [47]. Thus, for instance, sustained neural activation during the delay period is the mechanism that most attention has received from the modeling community to model working memory, and attractor networks have proven to be successful to account for this phenomenon (*e.g.* [46],[48]). Local inhibition is, in this context, necessary in order to enable stable states with spontaneous rates and an average synaptic long-term potentiation (LTP) in specific populations is required to give rise to local attractors with sustained high firing rates during the delay period. Moreover, working memory capacity, for instance, critically depends on the constraints that lateral inhibition imposes to the mnemonic activity [49],[50]. Similarly, in decision making, inhibition naturally mediates the competition between neural populations coding for the different alternatives and must be carefully set up for the network to operate in an appropriate regime. Thus, although it is indeed the interplay between collaborative (*i.e.* in this work mediated by means of the 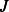 matrix) and competitive (*i.e.* in this work mediated by means of the 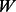 matrix) that gives rise to the emergence of the appropriate system dynamics, the role that inhibition plays appears to be especially critical as is the case, for instance, of establising the number of memories that can be actively maintained in working memory [51].

In this work, we have also attempted to provide insights into the cortical mechanisms underlying previous phenomenological models such as those by Blakeslee and McCourt [7] and Otazu et al. [9]. Notably, some of the constraints imposed in these models have emerged from the neuronal network structure and its associated prescribed dynamics. In particular, we have recovered the power function with slope 0.1 that was used to weigh the filters considered by Blakeslee and McCourt model [7] that is consistent with the shallow low-frequency fall-off of the suprathreshold CSF associated with the high-contrast stimuli employed, and have discussed some neural mechanisms underlying the emergence of perceptual images built from the same multi-scale and multi-orientation wavelet decomposition of the visual stimuli reported by Otazu et al. [9].

Finally, in the last years, the role of oscillations in multiple neurocognitive processes has received considerable attention. Interestingly, one of the pecularities of the proposed model is the oscillatory nature of its outputs. This, as argued by Li [35], is a relatively common feature of networks of excitatory and inhibitory neurons. The model has therefore the potential to explore the role that such oscillatory activity may have for encoding different aspects of BI. Indeed, Biederlack et al. [52] reported some suggestive evidence that rate enhancement and neuronal synchronization could contribute complementary codes of BI and further investigations in this area are certainly interesting.

A further extension of the model could certainly come from its generalization into color opponent space in order to reproduce chromatic induction effects, which would be important both from a fundamental perspective (*i.e.* gaining further understanding about color vision) but could also have an enormous impact in technological applications within the field of image processing.

Taken together, we propose a neurodynamical model of V1 from which BI emerges naturally. This model is embedded in a general framework to investigate contextual effects in V1 and, importantly, offers plausible explanations of the mechanisms yielding BI based on neural mechanisms. Finally, the model also makes specific firing rates and behavioural predictions and suggests how these can be related with manipulable experimental variables. In this sense, the study confirms the usefulness of computational neuroscience approaches to investigate neural processes and offers predictions which may be used to guide, in a principled way, the design of experiments in order to further explore BI.

## Materials and Methods

### Computational Model Description

The multiresolution wavelet decomposition is based on the *à trous* algorithm [53] (see detail in Text S2). The wavelet basis functions, or mother wavelets, are not strictly Gabor functions, but are smooth, symmetric, highly concentrated in both space and frequency, and have similar profile. This algorithm has two main advantages. First, it is undecimated (all the planes have the same resolution, independently of the spatial frequency they correspond to), which is a required property for the decomposition to be translation invariant. This point is of importance since, together with the invariance with respect to translation of the connectivity, it makes the whole system translation invariant. Furthermore, this decomposition allows every spatial frequency to be represented at each position representing a hypercolumn, in accordance with the architecture of the cortical integration region in monkey striate cortex [54].

The coefficients 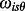 derived from a multiscale and multiorientation decomposition (Equation 1) are then half-wave rectified to preserve information regarding contrast polarity. The corresponding values 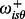 and 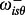 are then normalized in the range 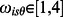 [35]. In the case of a static stimulus (resp. of a dynamic stimulus), the minimum and maximum of the multiresolution coefficients over all positions, all scales, and all orientations (resp. and all frames) are respectively set to the minimum and maximum values in that range. Then, all the minimum values (equal to 1) are set to 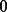. These normalized coefficients 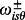, or 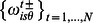, are then used to initialize the activity 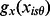 and serve as the input 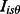 to the network that feeds the excitatory cells 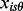. For static stimuli, the visual input 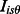 persists over time.

For simulating the dynamic of the model, we used a discrete time implementation. An Euler integration scheme of first order was used to numerically integrate the coupled differential equations describing the dynamics of the system. The time step was 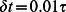, where 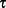 is the membrane time constant (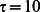 ms [35]). When considering a static stimulus, this input is constant over all the time membrane constants handled in the computation [31]. In the dynamic case, the relation between the frequency of oscillation 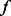 of a stimulus sinusoidally modulated in time and the temporal behavior of the model, which is dictated by its membrane time constant 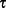, is computed as follows: during every time membrane constant 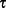, the stimulus undergoes a change corresponding to a 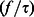-th part of a period.

## Supporting Information

**Supporting Information S1**. The computer code of the model is available at: http://www.cvc.uab.cat/xotazu/NeuroV1/BrightnessInduction/.

Text S1 **Supplementary Material.** Neurodynamical model of contextual influences mediated by intra-cortical interactions in V1 (following [35]). Proposed model parameters.
(PDF)

Text S2 **Supplementary Material.** Wavelet analysis.
(PDF)

Text S3 **Supplementary Material.** Supplementary results (Figure S1 in Text S3 and Figure S2 in Text S3).
(PDF)

Text S4 **Supplementary Material.** Frequency gain of the model (Figure S3 in Text S4).
(PDF)
