# Supplementary material for: A Neurodynamical Model of Brightness Induction in V1
Source: PLoS One. 2013 May 22;8(5):e64086. doi: 10.1371/journal.pone.0064086 (PMC3661450; doi:10.1371/journal.pone.0064086)
Supplement: Text S1 — Supplementary Material. Neurodynamical model of contextual influences mediated by intra-cortical interactions in V1 (following [35]). Proposed model parameters. (PDF) [file pone.0064086.s001.pdf]

Text S1. Neurodynamical model of contextual influences mediated by intra-cortical interactions in V1

Li's model parameters

In Li's original model [1], visual inputs are modeled at discrete spatial locations, where a V1 hypercolumn is characterized by K neuron units $[i,\,\theta]$ (each pair involving an excitatory and an inhibitory unit), with receptive fields (RFs) centered at position i and preferred orientation $\theta = \frac{k\pi}{K}$, for $k = 1,\, 2,\, \ldots\, K$. This, in fact, corresponds to a neural representation of an edge segment in which an excitatory and an inhibitory neuronal population are connected with each other. While the excitatory cells receive the visual input and their output quantify the response to the edge segment, the inhibitory cells are modeled as interneurons. The model assumes that the response from the excitatory neurons projects to higher areas whereas inhibitory neurons only provides feedback inhibition.
The input that the excitatory neurons associated with the spatial location i and preferred orientation θ receive from an edge (in the input image) with strength $\hat{I}_{i\beta}$ and orientation β is $I_{i\theta} = \hat{I}_{i\beta} \cdot \Phi(\theta-\beta)$, where $\Phi(\theta - \beta) = e^{-|\theta-\beta|/(\pi/8)}$. In the model, the excitatory and inhibitory cells have membrane potentials $x_{i\theta}$ and $y_{i\theta}$, respectively, and their outputs are obtained from sigmoid-like positive non-linear and non-decreasing functions $g_x(x_{i\theta})$ and $g_y(y_{i\theta})$, which represent the firing rates. A key aspect of the model is the connectivity it exhibits since this connectivity determines the dynamics of the neuronal system. The response of the model is determined by both its input image $\hat{I}_{i\beta}$ and the recurrent interactions. Based on neurophysiological observations (see references in [1]), contextual influences are mediated by horizontal connections. Two types of horizontal connections from bar $[j, \theta']$ to bar $[i, \theta]$ are considered: (i) those leading to monosynaptic excitation (represented by $J_{[i\theta, j\theta']}$), and (ii) dysnaptic connections, which are at the core of inhibition (represented by $W_{[i\theta,j\theta']}$). In fact, only those bars which have nearby RF centers and similar orientations are effectively connected. In Li's model, the membrane potentials describing the dynamics of the excitatory-inhibitory network obey the following differential equations:

$$\begin{cases} \dot{x}_{i\theta} = -\alpha_x x_{i\theta} - g_y(y_{i\theta}) - \sum_{\Delta\theta \neq 0} \psi(\Delta\theta) g_y(y_{i\theta+\Delta\theta}) + J_0 g_x(x_{i\theta}) \\ \qquad\qquad + \sum_{j \neq i, \theta'} J_{[i\theta,j\theta']} g_x(x_{j\theta'}) + I_{i\theta} + I_0, \\ \dot{y}_{i\theta} = -\alpha_y y_{i\theta} + g_x(x_{i\theta}) + \sum_{j\neq i,\theta'} W_{[i\theta,j\theta']} g_x(x_{j\theta'}) + I_c. \end{cases}$$

In these equations, $\psi(\Delta\theta) \leq 1$ represents the spread of the inhibitory activity within a hypercolumn, $J_0 g_x(x_{i\theta})$ models self-excitatory connections, I_c and I_0 correspond to background inputs (*i.e.* noise and inputs modeling the general and local normalization of activities). Depending on the visual stimuli, the system settles into an oscillatory state, and temporal averages of $g_x(x_{i\theta})$ over several oscillation cycles (about 12–24 membrane time constants) are used as the output of the model.

The model parameters are defined as follows:

$\alpha_x = \alpha_y = 1;\ K = 12;\ T_x = 1;\ L_y = 1.2;\ g_1 = 0.21;\ g_2 = 2.5$

$$g_x(x) = \begin{cases} 0 & \text{if } x < T_x \\ (x - T_x) & \text{if } T_x \le x \le T_x + 1 \\ 1 & \text{if } x \ge T_x \end{cases}$$

$$g_y(y) = \begin{cases} 0 & \text{if } y < 0 \\ g_1 y & \text{if } 0 \le y \le L_y \\ g_1 L_y + g_2(y - L_y) & \text{if } 0 < L_y \le y \end{cases}$$

$$\psi(\theta) = \begin{cases} 1 & \text{when } \theta = 0 \\ 0.8 & \text{when } |\theta| = \pi/K = 15^0 \\ 0.7 & \text{when } |\theta| = 2\pi/K = 30^0 \\ 0 & \text{otherwise} \end{cases}$$

$I_c = 1.0 + I_{\text{noise}}$
$I_0 = 0.85 + I_{\text{norm}} + I_{\text{noise}}$
$I_{\text{norm},i\theta} = -2.0 \left(\frac{\sum_{j \in S_i} \sum_{\theta'} g_x(x_{j\theta'})}{\sum_{j \in S_i} 1} \right)^2,$
$S_i = \{j | d(i,j) \le 2\}$, where $i = (i_1, i_2)$ and $j = (j_1, j_2)$ are considered as points with integer coordinates on a regular grid embedded in the real plane and d is the Euclidian distance $d(i,j) = \sqrt{(i_1 - j_1)^2 + (i_2 - j_2)^2}$.

$I_{\text{noise}} = \mathcal{N}(\bar{x}; \sigma_t, \sigma_x) = \mathcal{N}(0; 0.1, 0.1)$

$J_0 = 0.8$

$$J_{[i\theta, j\theta']} = \begin{cases} 0.126 \cdot e^{-(\beta/d)^2 - 2(\beta/d)^7 - d^2/90} & \text{if } (0 < d \le 10 \text{ and } \beta < \pi/2.69) \\ & \text{or } \big[(0 < d \le 10 \text{ and } \beta < \pi/1.1) \\ & \text{and } |\theta_1| < \pi/5.9 \text{ and } |\theta_1| < \pi/5.9\big] \\ 0 & \text{otherwise} \end{cases}$$

$$W_{[i\theta, j\theta']} = \begin{cases} 0 & \text{if } d = 0 \text{ or } d \ge 10 \text{ or } \beta < \pi/1.1 \\ & \text{or } |\Delta\theta| \ge \pi/3 \text{ or } |\theta_1| < \pi/11.999 \\ 0.14 \cdot (1 - e^{-0.4(\beta/d)^{1.5}}) e^{-(\Delta\theta/(\pi/4))^{1.5}} & \text{otherwise} \end{cases}$$

In the previous equations, $d = d(i,j)$, and θ_1, θ_2 are the angles between the edge elements at positions i and j and the line defined by $i - j$, with $|\theta_1| \le |\theta_2| \le \pi/2$. The sign of the angles is determined by the condition $|\theta_i| \le \pi/2$. Finally, $\beta = 2\theta_1 + 2\sin(|\theta_1 + \theta_2|)$, and $\Delta\theta = \theta - \theta'$ (with $|\theta - \theta'| \le \pi/2$).

Parameters of the model

In the model that we propose in this work, V1 hypercolumns are characterized by $S \times K$ neurons units $[i, s, \theta]$ (each pair involving an excitatory and an inhibitory unit), with RFs centered at i, preferred spatial frequency $f_s = 2^{-(s-1)} f_0$ cycles per image, where $s = 1, 2, ..., S$ and $f_0 \simeq N/10$ cycles per image (N is the size of the square grid of units), preferred orientation $\theta = 0, \pi/4, \pi/2, 3\pi/4$ ($K = 4$), also referred to in the main text as horizontal, first diagonal, vertical, and second diagonal orientations and denoted by (h, d_1, v, d_2), respectively.

The dynamic equations governing the behavior of the proposed model are:

$$\begin{cases} \dot{x}_{is\theta} = -\alpha_x x_{is\theta} - g_y(y_{is\theta}) - \sum_{\Delta s, \Delta\theta \neq 0} \psi(\Delta s, \Delta\theta) g_y(y_{is+\Delta s\theta+\Delta\theta}) + J_0 g_x(x_{is\theta}) \\ \qquad\qquad + \sum_{j\neq i, s', \theta'} J_{[is\theta, js'\theta']} g_x(x_{js'\theta'}) + I_{is\theta} + I_0, \\ \dot{y}_{is\theta} = -\alpha_y y_{is\theta} + g_x(x_{is\theta}) + \sum_{j\neq i, s', \theta'} W_{[is\theta, js'\theta']} g_x(x_{js'\theta'}) + I_c. \end{cases}$$

Below, we only describe the parameters that differ from those used in Li [1], which are described in the above section.

$K = 4$
$S = \log_2(N/16)$, where N is the size of the square grid ($N = 256$ for the static effects, $N = 128$ for the dynamical effect).
$\psi(\Delta s, \Delta\theta) = \lambda(\Delta s)\psi(\Delta\theta)$, where

$$\lambda(\Delta s) = \begin{cases} 1 & \text{when} \quad \Delta s = 0 \\ c & \text{when} \quad |\Delta s| = 1 \quad \text{(i.e. } \Delta s = s - s' \text{ for two neighboring scales } s \text{ and } s'\text{)} \\ 0 & \text{otherwise} \end{cases}$$

and $\psi(\Delta\theta) = \cos(\Delta\theta)^3$.
The value $c = 0$ corresponds to the case where there is no scale interaction in the model (perceptual channels of one octave). The results presented in this work corresponds to the value $c = 0.05$, corresponding to a slight mutual influence between neighboring channels.

$$I_{\text{norm}}(is\theta) = -2.0 \left(\frac{\sum_{j\in S_i} \sum_{\theta'} g_x(x_{js\theta'})}{\sum_{j\in S_i} 1} \right)^2$$

$J_{[is\theta, js'\theta']} = \lambda(s - s') J^s_{[i\theta, j\theta']}$, where

$$J^s_{[i\theta, j\theta']} = \begin{cases} 0.126 \cdot e^{-(\beta/d_s)^2 - 2(\beta/d_s)^7 - d_s^2/90} & \text{if } (0 < d_s \leq \Delta_s \text{ and } \beta < \pi/2.69) \\ & \text{or } \left[(0 < d_s \leq \Delta_s \text{ and } \beta < \pi/1.1) \right. \\ & \left. \text{and } |\theta_1| < \pi/5.9 \text{ and } |\theta_1| < \pi/5.9\right] \\ 0 & \text{otherwise} \end{cases}$$

$\Delta_s = 10 \cdot (2\epsilon)^{(s-1)}$, where $\epsilon = 1.1$
$d_s(i, j) = d(i, j)/(2\epsilon)^{(s-1)}$
and
$W_{[is\theta, js'\theta']} = \lambda(s - s') W^s_{[i\theta, j\theta']}$, where

$$W^s_{[i\theta, j\theta']} = \begin{cases} 0 & \text{if } d_s = 0 \text{ or } d_s \geq \Delta_s \text{ or } \beta < \pi/1.1 \\ & \text{or } |\Delta\theta| \geq \pi/3 \text{ or } |\theta_1| < \pi/11.999 \\ 0.14 \cdot (1 - e^{-0.4(\beta/d_s)^{1.5}}) e^{-(\Delta\theta/(\pi/4))^{1.5}} & \text{otherwise.} \end{cases}$$

References

1. Li Z (1999) Visual segmentation by contextual influences via intra-cortical interactions in the primary visual cortex. Network-Comp Neural 10: 187–212.
