# Supplementary material for: A Neurodynamical Model of Brightness Induction in V1
Source: PLoS One. 2013 May 22;8(5):e64086. doi: 10.1371/journal.pone.0064086 (PMC3661450; doi:10.1371/journal.pone.0064086)
Supplement: Text S2 — Supplementary Material. Wavelet analysis. (PDF) [file pone.0064086.s002.pdf]

Text S2. Multiscale and multiorientation decomposition

We use a shift-invariant discrete wavelet decomposition following [1] and adopt the *à trous* discrete wavelet transform algorithm [2]. Given an image $\mathbf{I}$, we construct a sequence of approximations $\{\mathbf{c}_s\}_{s=1,\ldots,S}$, with $\mathbf{c}_0 \equiv \mathbf{I}$ and $\mathbf{c}_1 = F_1(\mathbf{c_0})$, $\mathbf{c}_2 = F_2(\mathbf{c}_1)$, $\mathbf{c}_3 = F_3(\mathbf{c}_2)$, $\ldots$, where the $F_{-}(\cdot)$ are size-preserving transforms described below. To obtain the sequence, the algorithm performs successive convolutions with a filter obtained from an auxiliary function named scaling function [3]. For the sake of simplicity, we give a one-dimensional version of the decomposition. The use of a B_3 cubic spline for the scaling function leads to a convolution with the filter

$$h_1 = \frac{1}{16}\begin{pmatrix} 1 & 4 & 6 & 4 & 1 \end{pmatrix} . \quad \text{(S.1)}$$

Hence, we can write

$$\mathbf{c}_s \equiv F_s(\mathbf{c}_{s-1}) = \mathbf{c}_{s-1} \otimes h_s \ , \quad \text{(S.2)}$$

where $\otimes$ denotes the convolution operation. The filter h_s is obtained from h_{s-1} by doubling its size, *i.e.* $h_s = \uparrow h_{s-1}$, where $\uparrow$ means upsampling by introducing zeros between the coefficients in h_{s-1} (hence the name *à trous*). The wavelet planes are computed as the differences between two consecutive approximations $\mathbf{c}_{s-1}$ and $\mathbf{c}_s$,

$$\omega_s = \mathbf{c}_{s-1} - \mathbf{c}_s,\ s = 1, \ldots, S. \quad \text{(S.3)}$$

The reconstruction formula is

$$\mathbf{I} = \sum_{s=1}^{S} \omega_s + \mathbf{c}_S. \quad \text{(S.4)}$$

In this representation, the images $\mathbf{c}_s$ ($s = 0, \ldots, S$) are versions of the original image $I \equiv \mathbf{c}_0$ at increasing scales (*i.e.* decreasing resolution levels). The terms ω_s ($s = 1, \ldots, S$) are the multiresolution wavelet planes and $\mathbf{c}_n$ is a residual image. In this work, a diadic decomposition scheme is used. Thus, the spatial resolution of the original image $\mathbf{c}_0$ is twice that of $\mathbf{c}_1$, the spatial resolution of $\mathbf{c}_1$ is twice that of $\mathbf{c}_2$, and so on. Note, however, that all the consecutive approximations $\mathbf{c}_s$ (and wavelet planes ω_s) in this process have the same number of pixels as the original image (the decomposition is said to be undecimated). This is a consequence of the fact that the *à trous* algorithm is a non-orthogonal oversampled transform [4], which is equivalent to a bi-orthogonal wavelet transform [3].
The above decomposition can be generalized to several spatial directions by performing the convolutions along different orientations. In this work we used four orientations, namely horizontal, first diagonal, vertical, and second diagonal. In that case, the reconstruction formula can be written as

$$\mathbf{I} = \sum_{s=1}^{S} \sum_{\theta=h,d_1 v,d_2} \omega_{s\theta} + \mathbf{c}_S \ . \quad \text{(S.5)}$$

References

1. Starck JL, Murtagh F (1994) Image restoration with noise suppression using the wavelet transform. Astronomy and Astrophysics 288: 342-348.

2. Holschneider M, Tchamitchian P (1990) Les ondelettes en 1989. Springer Verlag.

3. Mallat S (1998) A wavelet tour of signal processing, second edition. Academic Press.

4. Vetterli M, Kovacevic J (1995) Wavelets and subband coding. Prentice Hall.
