# Supplementary material for: A Neurodynamical Model of Brightness Induction in V1
Source: PLoS One. 2013 May 22;8(5):e64086. doi: 10.1371/journal.pone.0064086 (PMC3661450; doi:10.1371/journal.pone.0064086)
Supplement: Text S3 — Supplementary Material. Supplementary results (Figure S1 in Text S3 and Figure S2 in Text S3). (PDF) [file pone.0064086.s003.pdf]

Text S3. Supplementary results

Relative spatial orientation. So far, the interactions between different orientations have not played a critical role in the results since the stimuli considered were not designed to probe their effect. However, the model does, by design, take into account orientation sensitivity. Thus, in order to explore the potential of the model, we have addressed the influence of relative orientation on BI. Figures S**1A** and **E** show two vertically oriented square gratings of the same spatial frequency on which a gray stripe has been superposed. The two figures only differ in the orientation of the gray stripe. In fact, Figure S**1A** is another instantiation of the White effect, whereas Figure S**1E** provides an illustration of a (square) GI effect. Similarly, the visual stimulus displayed in Figure S**1C** contains a vertically oriented square grating (as in Figures S**1A** and **E**) but the overlying stripe has an intermediate orientation, which is denoted as diagonal and corresponds to 45°.

As can be observed, brightness assimilation of the central feature takes place when it shares its main orientation with the surrounding features. Furthermore, the mean brightness value of the gray stripe becomes a function of the difference in its orientation and that of the background grating, which in fact varies from pure assimilation and zero amplitude oscillations in the case of stripe coaligned with the background, to brightness contrast (and increasing amplitude) as the difference in orientation between the two elements increases. In fact, this is a prediction of the model since, to the best of our knowledge, only the BI effects derived from the parallel and orthogonal orientations have been previously reported. Figures **S1B**, **D** and **F** show such predictions, which can be broadly interpreted by considering the connectivity of the model and the response of different neuronal populations to the visual stimuli. Indeed, the effect of the disynaptic inhibition is strongest when the stripe is coaligned with the background and for that orientation which is parallel to that of the stimuli (for the appropriate spatial frequency, *i.e.* that of the grating). Inhibition lessens with increasing differences in orientation for that spatial frequency and, in fact, nearly vanishes for orthogonal orientations, thus leading to larger amplitudes in the perceived luminance of the induced grating. Interestingly, brightness assimilation from the lateral flanks might appear as being the strongest effect among the three cases herein considered because of the non-periodic nature of the perceived luminance. However, the absolute differences regarding the actual stimulus are locally larger in the (orthogonal) GI effect.

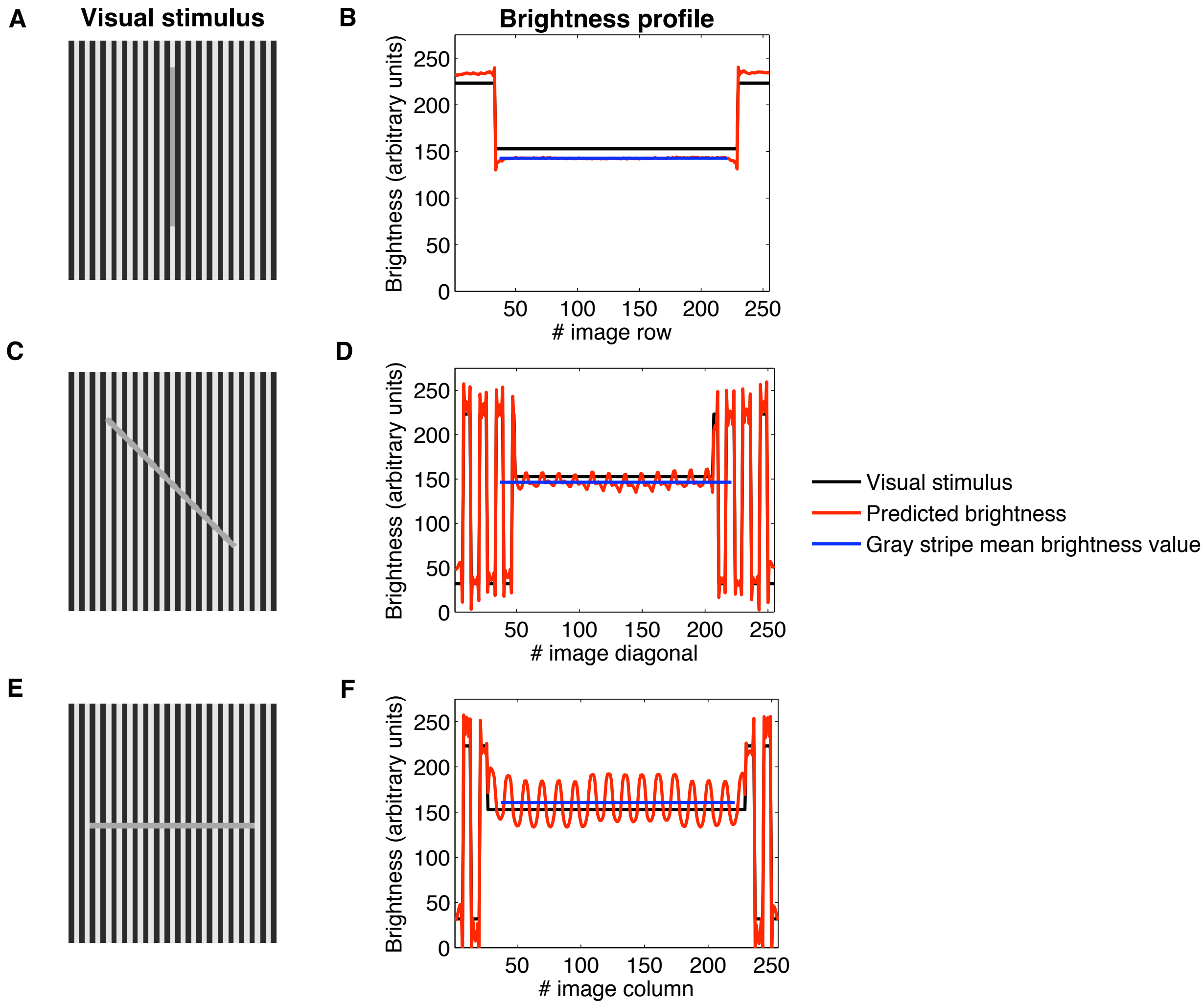

Figure S1. Effect of relative orientation on brightness assimilation. The vertical stripe (**A**) is perceived darker than the diagonal stripe (**C**), which, in turn, is perceived darker than the horizontal stripe (**E**). This is a consequence of both the non-periodic nature of the perceived signal in (**A**) and the transition from assimilation to brightness contrast observed when increasing the difference in orientation between the stripe and the background. (**B**,**D**,**F**) show that the mean firing rates predicted by the model agree with perception. The blue solid curve corresponds to the mean brightness value along the stripe, as predicted by the model.

Chevreul effect. In the Chevreul effect, a series of stripes whose luminance has a staircase profile is perceived as having a sawtooth profile. Figure S**2A** shows an example of this effect, whereas Figure S**2B** illustrates the successful prediction derived from the model. The operation of the model for the Chevreul and the Mach bands effects are, in fact, similar. Each edge produces a strong activity in the cell population which is sensitive to its orientation and frequency. This takes place for each polarity at one

side of the edge (*i.e.* right side for the bright-dark transition and left side of the edge for the dark-bright transition). As a result, the activity of the co-aligned cells at the edges that share the same orientation sensitivity is enhanced, thereby explaining the emergence of a sawtooth profile.

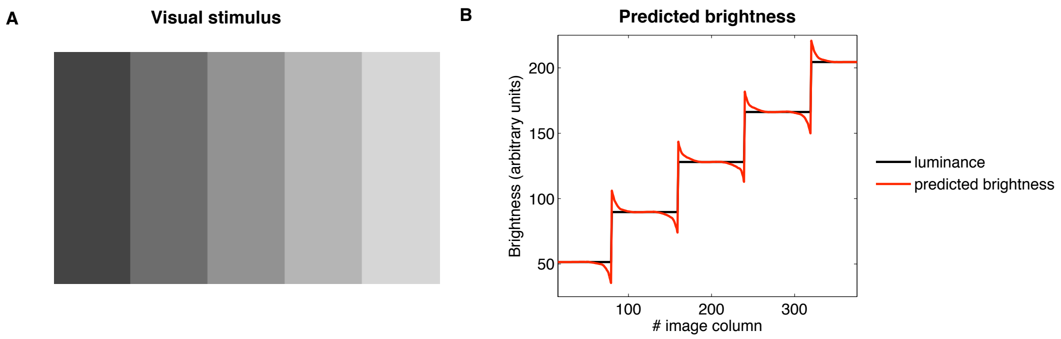

Figure S2. Chevreul effect. (**A**) Bands whose luminance is uniform do not appear uniform: areas near the vertical edges are perceived darker or brighter depending on the luminance of the neighboring bands.(**B**) The predicted mean firing rates agree with the fact that the brightness shows a sawtooth profile whereas the original luminance has a staircase profile.
