# Supplementary material for: A Neurodynamical Model of Brightness Induction in V1
Source: PLoS One. 2013 May 22;8(5):e64086. doi: 10.1371/journal.pone.0064086 (PMC3661450; doi:10.1371/journal.pone.0064086)
Supplement: Text S4 — Supplementary Material. Frequency gain of the model (Figure S3 in Text S4). (PDF) [file pone.0064086.s004.pdf]

Text S4. Frequency gain of the model

We computed the gain of the model for sinusoidal gratings with different spatial frequencies ranging between one cycle per image to the highest frequency (128 cycles per image for 256×256 images). Figure S**3** shows that in $\log - \log$ coordinates the gain function can be approximated by a line of slope 0.092. This indicates that the frequency weighting function is similar to a power law function with a slope close to 0.1. This last value was used in the DOG and ODOG models and is consistent with the shallow low-frequency fall-off of the suprathreshold CSF [1–3].

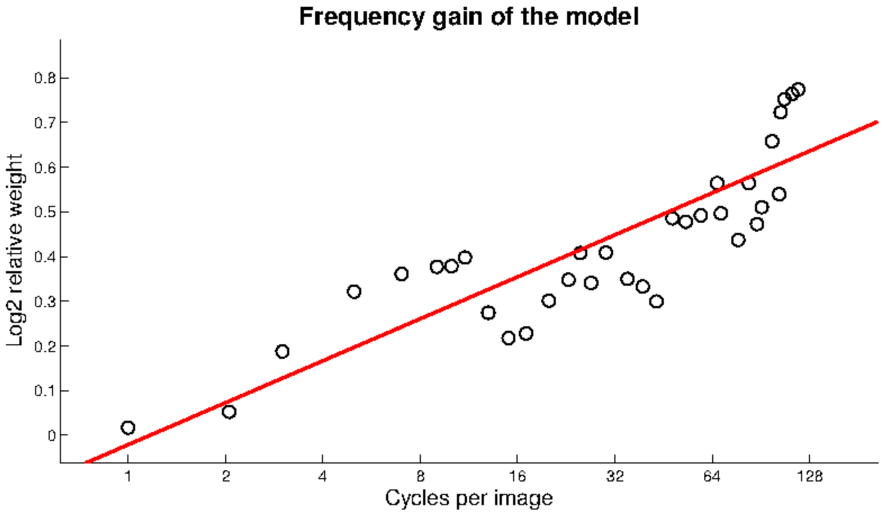

Figure S3. Frequency gain of the model. The (relative) gain was plotted against frequency in $\log_2 - \log_2$ coordinates (black circles). The points are distributed around a line of equation $\log_2(\text{gain}) = C + 0.092 \cdot \log_2(f)$ (least square, $r^2 = 0.85$, $p < 10^{-5}$, rms error $= 0.09$), where 'gain' is the relative weight and f is the frequency in cycles per image.

References

1. Blakeslee B, McCourt M (2011) Similar mechanisms underlie simultaneous brightness contrast and grating induction. Vision Res 37: 2849–2869.

2. Blakeslee B, McCourt M (1999) A multiscale spatial filtering account of the white effect, simultaneous brightness contrast and grating induction. Vision Res 39: 4361–4377.

3. Blakeslee B, McCourt M (2004) A unified theory of brightness contrast and assimilation incorporating oriented multiscale spatial filtering and contrast normalization. Vision Res 44: 2483–2503.
